# Supplementary material for: Differential cortical layer engagement during seizure initiation and spread in humans
Source: Nat Commun. 2024 Jun 17;15:5153. doi: 10.1038/s41467-024-48746-8 (PMC11183216; doi:10.1038/s41467-024-48746-8)
Supplement: Supplementary file 1 — Supplementary Information [file 41467_2024_48746_MOESM1_ESM.pdf]

Patient	Localization of the ictal onset zone	Etiology of the epilepsy	Epilepsy	Laminar electrode inside the SOZ	Laminar electrode outside the SOZ (distance from the ictal onset zone in cm)	Additional laminar electrode outside the SOZ (distance from the ictal onset zone in cm)	Number of seizures
1	Frontal (parasagittal), right	Cryptogenic	Partial complex	No	Surface to deep supra-granular pattern (5 cm)	No	9
2	Temporal (non-mesial), right	Porencephalic cyst	Partial complex	Infragranular pattern	Bad signal quality (6.5 cm)	No	5
3	Temporal (non-mesial), right	Focal cortical dysplasia	Partial complex	No	Surface to deep supra-granular pattern (5.5 cm)	Surface to deep supra-granular pattern (7 cm)	3
4	Temporal (mesial), right	Hippocampal sclerosis	Partial complex	No	Surface to deep supra-granular pattern (5 cm)	No	2
5	Frontal, right	Oligodendroglioma	Partial complex	Granular & infra granular parallele pattern	Bad signal quality (5.5 cm)	No	1
6	Temporal (mesial), right	Hippocampal sclerosis	Partial complex	Granular & infra granular parallele pattern	Surface to deep supra-granular pattern (6 cm)	No	1
7	Temporal (non-mesial), left	Cryptogenic	Partial complex	No	Atypical (6 cm)	Atypical (6.5 cm)	2
8	Occipital, right	Cryptogenic	Partial complex	Granular & infra granular parallele pattern	No obvious propagation (7cm)	No	1
9	Temporal (non-mesial), right	Focal cortical dysplasia	Partial complex	No	Surface to deep supra-granular pattern (5 cm)	No	3
10	Frontal, right	Focal cortical dysplasia	Partial complex	Infragranular pattern	Surface to deep supra-granular pattern (6.5 cm)	No	3

Table S1: description of the patients and main findings from the laminar recordings
