# Supplementary material for: Differential cortical layer engagement during seizure initiation and spread in humans
Source: Nat Commun. 2024 Jun 17;15:5153. doi: 10.1038/s41467-024-48746-8 (PMC11183216; doi:10.1038/s41467-024-48746-8)
Supplement: Supplementary file 3 — Reporting Summary [file 41467_2024_48746_MOESM3_ESM.pdf]

nature portfolio

Corresponding author(s): Pierre Bourdillon

Last updated by author(s): Mar 18, 2024

Reporting Summary

Nature Portfolio wishes to improve the reproducibility of the work that we publish. This form provides structure for consistency and transparency in reporting. For further information on Nature Portfolio policies, see our Editorial Policies and the Editorial Policy Checklist.

Statistics

For all statistical analyses, confirm that the following items are present in the figure legend, table legend, main text, or Methods section.

n/a	Confirmed	
☐	☒	The exact sample size (*n*) for each experimental group/condition, given as a discrete number and unit of measurement
☒	☐	A statement on whether measurements were taken from distinct samples or whether the same sample was measured repeatedly
☐	☒	The statistical test(s) used AND whether they are one- or two-sided<br>*Only common tests should be described solely by name; describe more complex techniques in the Methods section.*
☒	☐	A description of all covariates tested
☒	☐	A description of any assumptions or corrections, such as tests of normality and adjustment for multiple comparisons
☐	☒	A full description of the statistical parameters including central tendency (e.g. means) or other basic estimates (e.g. regression coefficient) AND variation (e.g. standard deviation) or associated estimates of uncertainty (e.g. confidence intervals)
☐	☒	For null hypothesis testing, the test statistic (e.g. *F*, *t*, *r*) with confidence intervals, effect sizes, degrees of freedom and *P* value noted<br>*Give P values as exact values whenever suitable.*
☒	☐	For Bayesian analysis, information on the choice of priors and Markov chain Monte Carlo settings
☒	☐	For hierarchical and complex designs, identification of the appropriate level for tests and full reporting of outcomes
☒	☐	Estimates of effect sizes (e.g. Cohen's *d*, Pearson's *r*), indicating how they were calculated

Our web collection on statistics for biologists contains articles on many of the points above.

Software and code

Policy information about availability of computer code

Data collection	Na
Data analysis	For this study we used Matlab 2021b including fieldtrip toolbox, Jupyter, scikit-learn. Code available on upon request.

For manuscripts utilizing custom algorithms or software that are central to the research but not yet described in published literature, software must be made available to editors and reviewers. We strongly encourage code deposition in a community repository (e.g. GitHub). See the Nature Portfolio guidelines for submitting code & software for further information.

Data

Policy information about availability of data

All manuscripts must include a data availability statement. This statement should provide the following information, where applicable:

- Accession codes, unique identifiers, or web links for publicly available datasets
- A description of any restrictions on data availability
- For clinical datasets or third party data, please ensure that the statement adheres to our policy

As data were collected in patient undergoing iEEG for medical purpose, the IRB of our institution did not permit to have them stored outside the hospital server. Nevertheless they could be shared on request if the request is scientificaly justified.

Research involving human participants, their data, or biological material

Policy information about studies with human participants or human data. See also policy information about sex, gender (identity/presentation), and sexual orientation and race, ethnicity and racism.

Reporting on sex and gender	Not reported
Reporting on race, ethnicity, or other socially relevant groupings	Not reported
Population characteristics	The characteristics of the population such as age or sex have not been identified as relevant in the scientific literature concerning cortical electrophysiological studies. In accordance with our policy and ethical recommendations, only information necessary and justified by the research objective and solid scientific hypotheses can be extracted from the medical records (in this case, the cause of epilepsy as well as the variables reported in Table S1)
Recruitment	Participation in the study was offered to all patients scheduled for phase 2 pre-surgical evaluation and for whom surgical implantation of laminar electrodes was feasible.
Ethics oversight	Partners Health Care IRB (currently Massachusetts General Brigham IRB), the New York University Medical Center IRB, and the Hungarian Medical Scientific Council

Note that full information on the approval of the study protocol must also be provided in the manuscript.

Field-specific reporting

Please select the one below that is the best fit for your research. If you are not sure, read the appropriate sections before making your selection.

☒ Life sciences ☐ Behavioural & social sciences ☐ Ecological, evolutionary & environmental sciences

For a reference copy of the document with all sections, see nature.com/documents/nr-reporting-summary-flat.pdf

Life sciences study design

All studies must disclose on these points even when the disclosure is negative.

Sample size	As this was an exploratory study, it was not possible to anticipate what difference to show. The number of subjects participating in this exploratory study was determined by the logistical capacity for electrode implantation.
Data exclusions	There was no exclusion of data.
Replication	This study shows individual comparisons (each subject being able to serve as their own control during the implantation of multiple electrodes), where inter-subject replication was performed. Replication could indeed be possible but would require ethical justification as it would involve implanting laminar intracerebral electrodes again in human patients.
Randomization	Randomization is a technique aimed at comparing an intervention between two similar groups. Since this study is non-interventional and purely descriptive, randomization is not an appropriate methodology
Blinding	Blinding is a technique aimed at minimizing biases when comparing an intervention between two groups. Since this study is non-interventional and purely descriptive, blinding is not an appropriate methodology.

Behavioural & social sciences study design

All studies must disclose on these points even when the disclosure is negative.

Study description	Na
Research sample	Na
Sampling strategy	Na
Data collection	Na
Timing	Na
Data exclusions	Na

Non-participation	Na
Randomization	Na

Ecological, evolutionary & environmental sciences study design

All studies must disclose on these points even when the disclosure is negative.

Study description	Na
Research sample	Na
Sampling strategy	Na
Data collection	Na
Timing and spatial scale	Na
Data exclusions	Na
Reproducibility	Na
Randomization	Na
Blinding	Na

Did the study involve field work? ☐ Yes ☒ No

Field work, collection and transport

Field conditions	Na
Location	Na
Access & import/export	Na
Disturbance	Na

Reporting for specific materials, systems and methods

We require information from authors about some types of materials, experimental systems and methods used in many studies. Here, indicate whether each material, system or method listed is relevant to your study. If you are not sure if a list item applies to your research, read the appropriate section before selecting a response.

Materials & experimental systems

n/a	Involved in the study	
☒	☐	Antibodies
☒	☐	Eukaryotic cell lines
☒	☐	Palaeontology and archaeology
☒	☐	Animals and other organisms
☒	☐	Clinical data
☒	☐	Dual use research of concern
☒	☐	Plants

Methods

n/a	Involved in the study	
☒	☐	ChIP-seq
☒	☐	Flow cytometry
☒	☐	MRI-based neuroimaging

Antibodies

Antibodies used	Na
Validation	Na

Eukaryotic cell lines

Policy information about cell lines and Sex and Gender in Research

Cell line source(s)	Na
Authentication	Na
Mycoplasma contamination	Na
Commonly misidentified lines (See ICLAC register)	Na

Palaeontology and Archaeology

Specimen provenance	Na
Specimen deposition	Na
Dating methods	Na

☐ Tick this box to confirm that the raw and calibrated dates are available in the paper or in Supplementary Information.

Ethics oversight	Na

Note that full information on the approval of the study protocol must also be provided in the manuscript.

Animals and other research organisms

Policy information about studies involving animals; ARRIVE guidelines recommended for reporting animal research, and Sex and Gender in Research

Laboratory animals	Na
Wild animals	Na
Reporting on sex	Na
Field-collected samples	Na
Ethics oversight	Na

Note that full information on the approval of the study protocol must also be provided in the manuscript.

Clinical data

Policy information about clinical studies

All manuscripts should comply with the ICMJE guidelines for publication of clinical research and a completed CONSORT checklist must be included with all submissions.

Clinical trial registration	Na
Study protocol	IRB approved
Data collection	IRB approved
Outcomes	Na

Dual use research of concern

Policy information about dual use research of concern

Hazards

Could the accidental, deliberate or reckless misuse of agents or technologies generated in the work, or the application of information presented in the manuscript, pose a threat to:

No	Yes	
☒	☐	Public health
☒	☐	National security
☒	☐	Crops and/or livestock
☒	☐	Ecosystems
☒	☐	Any other significant area

Experiments of concern

Does the work involve any of these experiments of concern:

No	Yes	
☒	☐	Demonstrate how to render a vaccine ineffective
☒	☐	Confer resistance to therapeutically useful antibiotics or antiviral agents
☒	☐	Enhance the virulence of a pathogen or render a nonpathogen virulent
☒	☐	Increase transmissibility of a pathogen
☒	☐	Alter the host range of a pathogen
☒	☐	Enable evasion of diagnostic/detection modalities
☒	☐	Enable the weaponization of a biological agent or toxin
☒	☐	Any other potentially harmful combination of experiments and agents

Plants

Seed stocks	Na
Novel plant genotypes	Na
Authentication	Na

ChIP-seq

Data deposition

☐ Confirm that both raw and final processed data have been deposited in a public database such as GEO.

☐ Confirm that you have deposited or provided access to graph files (e.g. BED files) for the called peaks.

Data access links<br>*May remain private before publication.*	Na
Files in database submission	Na
Genome browser session<br>(e.g. UCSC)	Na

Methodology

Replicates	Na
Sequencing depth	Na
Antibodies	Na
Peak calling parameters	Na
Data quality	Na
Software	Na

Flow Cytometry

Plots

Confirm that:

☐ The axis labels state the marker and fluorochrome used (e.g. CD4-FITC).

☐ The axis scales are clearly visible. Include numbers along axes only for bottom left plot of group (a 'group' is an analysis of identical markers).

☐ All plots are contour plots with outliers or pseudocolor plots.

☐ A numerical value for number of cells or percentage (with statistics) is provided.

Methodology

Sample preparation	Na
Instrument	Na
Software	Na
Cell population abundance	Na
Gating strategy	Na

☐ Tick this box to confirm that a figure exemplifying the gating strategy is provided in the Supplementary Information.

Magnetic resonance imaging

Experimental design

Design type	Na
Design specifications	Na
Behavioral performance measures	Na

Acquisition

Imaging type(s)	Na
Field strength	Na
Sequence & imaging parameters	Na
Area of acquisition	Na

Diffusion MRI ☐ Used ☒ Not used

Preprocessing

Preprocessing software	Na
Normalization	Na
Normalization template	Na
Noise and artifact removal	Na
Volume censoring	Na

Statistical modeling & inference

Model type and settings	Na
Effect(s) tested	Na

Specify type of analysis: ☐ Whole brain ☐ ROI-based ☐ Both

Statistic type for inference<br>(See Eklund et al. 2016)	Na
Correction	Na

Models & analysis

n/a	Involved in the study
☒	☐ Functional and/or effective connectivity
☒	☐ Graph analysis
☒	☐ Multivariate modeling or predictive analysis

Functional and/or effective connectivity	Na
Graph analysis	Na
Multivariate modeling and predictive analysis	Na
